# Short-term effect of temperature on cause-specific, sex-specific, and age-specific ambulance dispatches in Czechia: a nationwide time-series analysis

**DOI:** 10.1093/ije/dyaf051

**Published:** 2025-05-28

**Authors:** Tomáš Janoš, Joan Ballester, Raúl F Méndez-Turrubiates, Pavel Čupr, Hicham Achebak

**Affiliations:** RECETOX, Faculty of Science, Masaryk University, Brno, Czech Republic; ISGlobal, Barcelona, Spain; ISGlobal, Barcelona, Spain; ISGlobal, Barcelona, Spain; RECETOX, Faculty of Science, Masaryk University, Brno, Czech Republic; ISGlobal, Barcelona, Spain; Inserm, France Cohortes, Paris, France

**Keywords:** ambulance, heat, cold, heat waves, temperature, climate change, emergency

## Abstract

**Background:**

Although several studies have investigated temperature-related mortality and morbidity, only a little is known about the short-term effects of temperature on ambulance dispatches. We aimed to conduct the first nationwide analysis of the association between temperatures and ambulance dispatches in Europe, including, for the first time, a detailed description of age-specific risks for 10-year age groups.

**Methods:**

We collected daily data on ambulance dispatches and climate (i.e. temperature and relative humidity) for each district of Czechia (*n* = 77) during 2010–19. We estimated the relationship for each district by using a quasi-Poisson regression with distributed lag non-linear models. We then applied a multilevel multivariate random-effects meta-analysis to derive regional and countrywide average associations and calculated the burden of ambulance dispatches that was attributable to non-optimum temperatures.

**Results:**

The susceptibility to low (high) temperatures increased (decreased) with age, except for the youth (<20 years), for whom the risks for both heat and cold were the highest. High temperatures contributed slightly to the risk of ambulance dispatches due to respiratory and cardiovascular causes, while the contribution of low temperatures was substantial. The overall ambulance dispatches burden that was attributable to non-optimum temperatures (optimum temperature = 7.9°C) was 3.55% (95% eCI: 3.43 to 3.67), with a predominant contribution of heat [2.32% (95% eCI: 2.15 to 2.46)] compared with cold [1.23% (95% eCI: 1.16 to 1.30)].

**Conclusion:**

This data can be used as an early-warning indicator for temperature impacts, especially among vulnerable population subgroups, such as children, adolescents, and young adults. This evidence has important implications for healthcare system preparedness and management, and for the projections of climate change health impacts.

Key Messages

- Non-optimum temperatures increase the risk of ambulance dispatches, with most of the burden attributable to heat.
- Young subpopulations, such as children, adolescents, and young adults, are the most vulnerable.
- These findings provide important evidence for better management of existing medical and emergency resources.

## Introduction

An accelerated rise in global average temperatures resulted in the hottest year on record in 2023, with global average temperatures that were 1.48°C warmer than in the pre-industrial climate.^1^ Moreover, the temperature increase for Europe is ∼1°C larger than the corresponding increase for the globe as a whole.^2^ In this context, Europe has emerged as a major climatic hotspot,^3^ with Czechia experiencing the most significant increase in the exposure to heatwaves within the continent, surpassing 200% in the 2010s compared with the 2000s.^4^

Both high and low ambient temperatures are leading environmental risk factors,^5^ with substantial health impacts among European populations.^6^^,^^7^ While the majority of epidemiological studies have investigated temperature-related mortality^6–10^ and hospital admissions,^11–14^ only a little is known about the effects of temperature on ambulance dispatches. The limited evidence available thus far suggests an increased risk of ambulance dispatches due to both cold^15–20^ and hot^15^^,^^17^^,^^19–23^ temperatures, with a predominant contribution of cold to the overall temperature-related ambulance dispatches burden.^16^^,^^20^^,^^24^^,^^25^ However, understanding the effect of temperature on ambulance service demands remains incomplete, as most current studies have been limited to urban populations^16^^,^^17^^,^^19^^,^^21^^,^^23^ and conducted in countries with distinct climate and socioeconomic conditions.^15^^,^^20^^,^^25^

Ambulance dispatches data can offer timely health information, serving as a crucial source of evidence on the early effects of moderate and extreme temperatures.^26^ Utilization of such data might be useful for the ability to conduct more targeted prevention measures, for better management of existing medical and emergency resources, for the evaluation of the effectiveness of existing early-warning systems,^27^ or even for the development of a new generation of early-warning systems that can be used not only by emergency medical services for their preparedness efforts, but also by the general public.^19^

To the best of our knowledge, the risk of increased ambulance dispatches due to non-optimum temperatures has not been explored at the national level in Europe to date. In addition, the scarcity of evidence across population subgroups (e.g. by sex, age, cause) makes it challenging to identify vulnerable populations and develop or strengthen long-term prevention and adaptation plans for emergency medical services. To address these knowledge gaps, this study aimed to estimate for the first time the association between non-optimum temperatures and ambulance dispatches in the districts of Czechia. Moreover, we quantified the burden of ambulance dispatches that was attributable to hot and cold temperatures, and conducted stratified analyses by sex, age, and cause of disease to identify potentially vulnerable population subgroups. Our findings have important implications for emergency medical services, public health and resource management, and health adaptation policies, aiming to effectively address the adverse impacts of climate change.

## Methods

### Data sources

We collected daily counts of all-cause, cardiovascular, and respiratory ambulance dispatches, disaggregated by sex, 10-year age categories (0–9, 10–19, …, 70–79, ≥80 years), and district of residence (77 units) within Czechia (pop. 10.9 million in 2023). The data span from 1 January 2010 to 31 December 2019, and were obtained from the Institute of Health Information and Statistics of the Czech Republic. We note that the dataset includes only dispatches to patients (e.g. ambulances that were transporting medical equipment are not covered), regardless of the outcome of the dispatch (on-site treatment, transport to a hospital, death). Daily gridded (0.10° × 0.10°) observations of daily mean 2-metre temperatures (°C) and daily mean relative humidity (%) were obtained from E-OBS (version 24.0e) of the European Climate Assessment and Dataset (ECA&D)^28^ and transformed into district estimates by weighting the values with population gridded (1 km^2^) counts for the year 2011 obtained from Eurostat.^29^

### Statistical analysis

In the first step, we estimated the cause-, sex-, and age-specific temperature–ambulance dispatche associations in each of the 77 districts of the Czech Republic by using a quasi-Poisson regression in combination with distributed lag non-linear models. Specifically, the model included: (i) an intercept, (ii) a categorical variable of the day of the week to take into account the weekly cycle in ambulance dispatches, (iii) a binary variable to control for national public holidays, (iv) a natural cubic spline of time with 8 degrees of freedom (df) per year to control for seasonal and long-term trends in ambulance dispatches, (v) a natural cubic spline with 4 df to adjust for relative humidity, and (vi) a cross-basis function of temperature produced by DLNM^30^ to describe the non-linear and delayed associations between temperature and ambulance dispatches. The baseline model equation can be written as follows:


log⁡(μ)=α+dow+nph+ns(time, 8 df per year)+ns(humidity)+cb(temperature)

where *µ* denotes the series of daily ambulance dispatches counts; *α* represents the intercept; *dow* represents the day of the week; *nph* represents national public holidays; *ns* represents the natural cubic spline; and *cb* represents the cross-basis function. The exposure–response function of the cross basis was modelled by using a natural cubic spline with one internal knot placed at the 50th percentile of the daily mean temperature distribution and the lag–response function was modelled by using a natural cubic spline with an intercept and three internal knots placed at equally spaced values in the log scale for ≤21 days. We used the model configuration that best fitted the data based on a quasi-likelihood version of the Akaike information criterion (Q-AIC) (see Supplementary Table S1).

Further, to examine any additional risk (i.e. added effect) of heatwaves on ambulance dispatches due to the persistence of extreme temperatures over a period of days, we included in the baseline model an indicator of heatwave days. To understand the characteristics of the lag effects of heat waves on ambulance dispatches, a natural cubic spline with 4 df was used to capture the distributed lag effect over time for ≤10 days. We placed two internal knots at equally spaced log values of lag, plus intercept. We defined a heat wave as a set of at least 2, 3, or 4 consecutive days with temperatures higher than the district-specific 95th percentile of the daily mean temperature distribution. To further examine the impacts of the intensity of heatwaves on ambulance dispatches, we used daily mean temperatures of the 90th and 97.5th percentiles as heatwave thresholds in combination with duration of ≥2 consecutive days.

In the second step, we used a univariate (for heatwaves) and multivariate (for temperature) multilevel meta-analysis^31^ to pool the district-specific estimates obtained in the previous stage, thus obtaining the average associations across the country. Residual heterogeneity was tested and quantified by usingthe multivariate extension of the Cochran Q test and the *I*^2^ statistic, respectively. Estimates were reported as relative risks (RRs) by using the minimum ambulance dispatch temperature (MADT), which corresponds to the optimum temperature, as the centring temperature. We also used the meta-analysis to derive the best linear unbiased predictions in each district, which were also used in the quantification of the ambulance dispatch burden that was attributable to temperatures in the following step.

In the last step, the RR associated with the exposure–response associations in each district were transformed into attributable fractions of ambulance dispatches due to heat and cold days by following the methodology described by Gasparrini and Leone.^32^ Heat and cold days were defined as days with temperatures higher and lower than the minimum ambulance dispatches temperature. We further separated these days into moderate and extreme parts by defining extreme heat (cold) as temperatures higher (lower) than the 97.5th (2.5th) district-specific temperature percentile. Finally, 95% empirical Confidence Intervals (eCIs) of attributable ambulance dispatches were calculated through Monte Carlo simulations, assuming that the coefficients have a multivariate normal distribution.

The modelling choices were thoroughly tested in several sensitivity analyses by varying the number of df to control for the seasonal and long-term trends, the number and placement of knots in the exposure–response function, the number of knots in the lag–response function, and the number of lag days.

All statistical analyses and visualizations were performed via R software (version 4.3.2) using the packages *splines*, *dlnm* (for the first-stage regression), *mixmeta* (for the second-stage meta-analysis), and *tmap*.

## Results

The study analysed a total of 8 634 643 ambulance dispatches between 2010 and 2019 in the 77 districts of Czechia, of which 49.8% corresponded to women. Individuals in the age category 80+ years accounted for the majority of ambulance dispatches (20.9%), followed by age categories 70–79 years (18.2%) and 60–69 years (16.0%). Cardiovascular and respiratory diseases represented 16.0% and 3.7% of the total ambulance dispatches, respectively. The distribution of ambulance dispatches counts by sex, age, specific diseases, and year of the study are reported in Supplementary Table S2. The daily mean population-weighted temperature had an average value of 9.2°C, with district-specific averages ranging from 7.3°C in Jablonec nad Nisou to 10.8°C in Prague. Descriptive statistics of the daily mean population-weighted temperature during the study period, as well as spatial distribution, are provided in Supplementary Table S3 and Supplementary Fig. S1.

**Figure 1.**
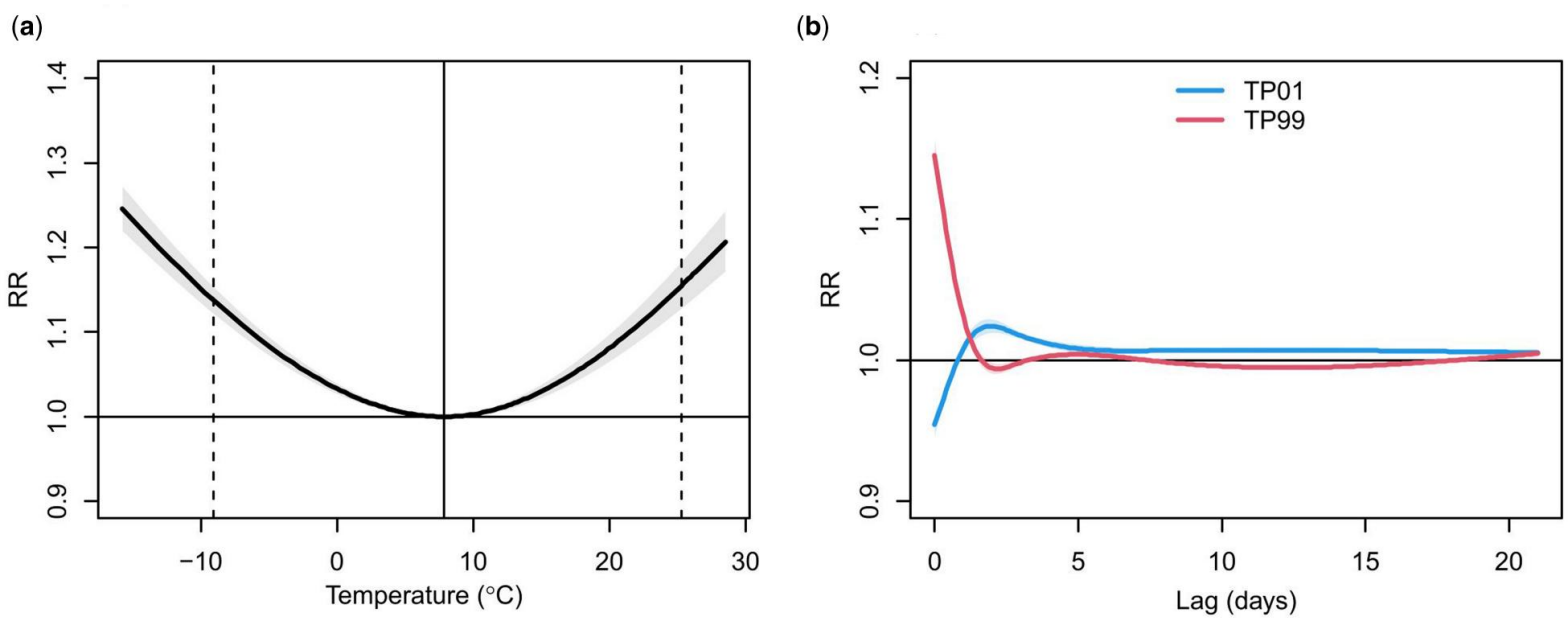
The overall cumulative exposure–response (A) and the predictor-specific lag–response (B) associations between temperature and ambulance dispatches for the overall population. RR, relative risk. TP01, temperature percentile 1. TP99, temperature percentile 99. Dashed vertical lines denote the 1st and 99th temperature percentiles. Solid vertical line denotes minimum ambulance dispatches temperature.

**Figure 2.**
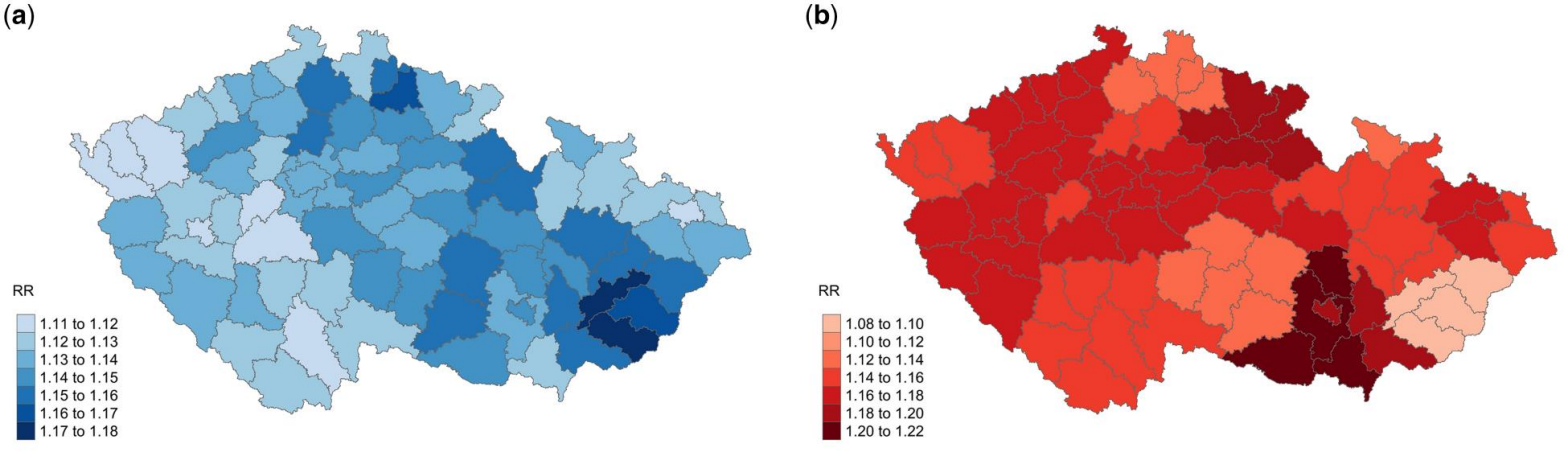
Spatial variability in relative risk of ambulance dispatches at the 1st (cold) (A) and 99th (heat) (B) temperature percentiles for the overall population. RR, relative risk.

The overall temperature–ambulance dispatches association and the predictor-specific lag–response association are illustrated in Fig. 1. The exposure–response curve exhibited a rather symmetric ‘U’ shape with a MADT of 7.9°C (45th temperature percentile) and a non-linear albeit smooth increase in the risk of ambulance dispatches for temperatures above and below this optimum temperature (Fig. 1a). The effect of extreme cold (i.e. 1st temperature percentile) was delayed (positive after a few days of exposure) and lasted for ≤3 weeks whereas the effect of extreme heat (i.e. 99th temperature percentile) was stronger, immediate, and short-lasting (2 days) (Fig. 1b). The multivariate Cochran Q test for heterogeneity was not significant (*P*-value 0.12) and the *I*^2^ statistic implied only a minor amount of residual heterogeneity for temperature–ambulance dispatches association (12.2%). The spatial heterogeneity in cold- and heat-related RRs of ambulance dispatches can be seen in Fig. 2.

**Figure 3.**
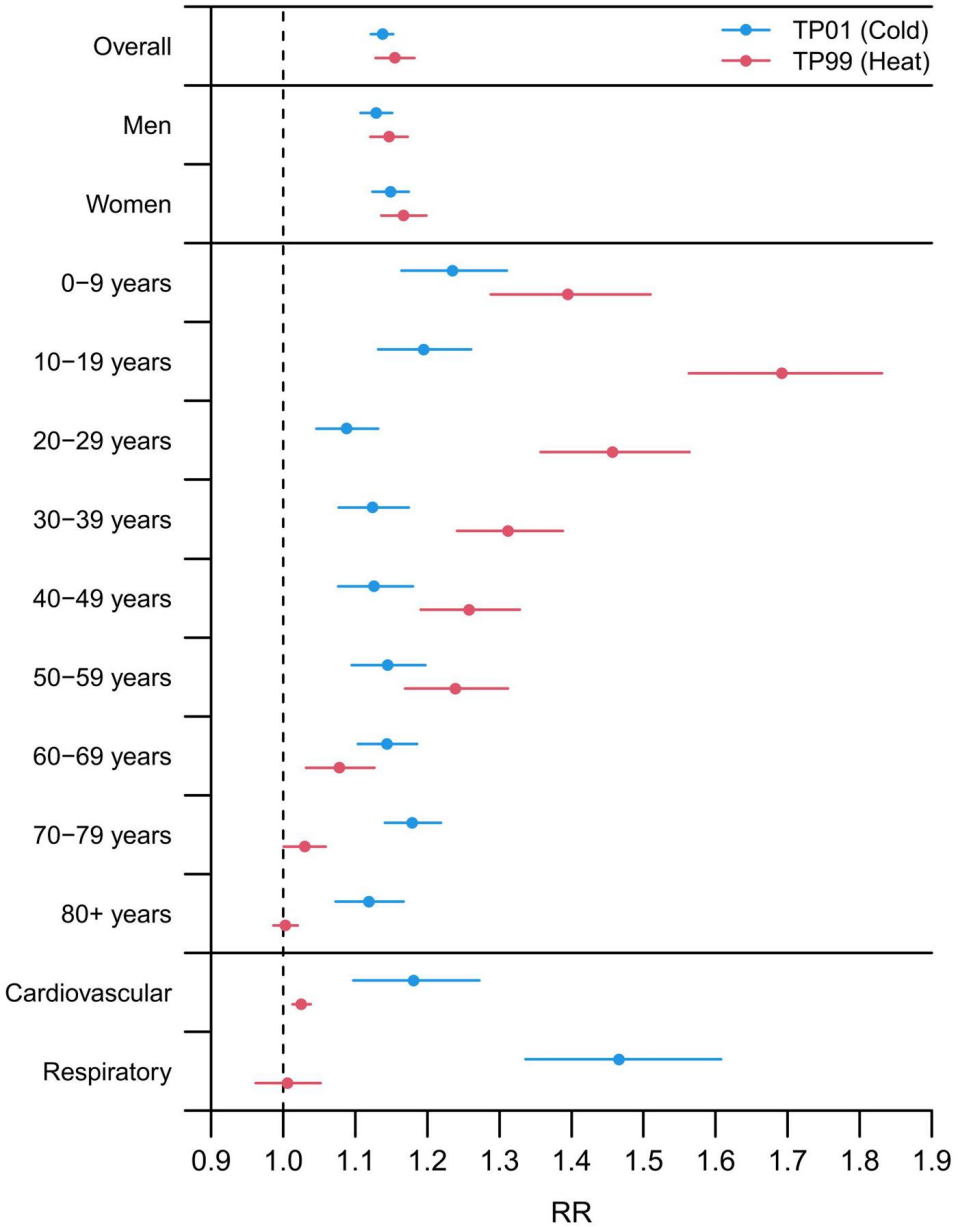
Relative risk of ambulance dispatches at the 1st (cold) and 99th (heat) temperature percentile. RR, relative risk. TP01, temperature percentile 1. TP99, temperature percentile 99. Numerical information is reported in Supplementary Table S4.

Figure 3 depicts the risk of ambulance dispatches associated with cold and heat stratified by sex, age, and cause of dispatch. We did not find any evidence for sex differences for both cold and heat. On the other hand, the risk of ambulance dispatches varied distinctly with age, with more pronounced differences in the effects of heat. The heat-related risk was highest among young people (≤29 years) and then declined during the rest of life, with a non-significant effect of heat in the oldest age category (80+ years). In a similar way, the risk of ambulance dispatches due to cold was the highest in childhood and adolescence, dropped down in young adults, and then slowly rose in older ages. Results for cause-specific ambulance dispatches showed limited evidence of the risk due to heat, but cold considerably increased the risk of ambulance dispatches from cardiovascular causes [RR: 1.181 (95% eCI: 1.097 to 1.272)] and even more extensively from respiratory causes [1.466 (1.336 to 1.608)].

**Figure 4.**
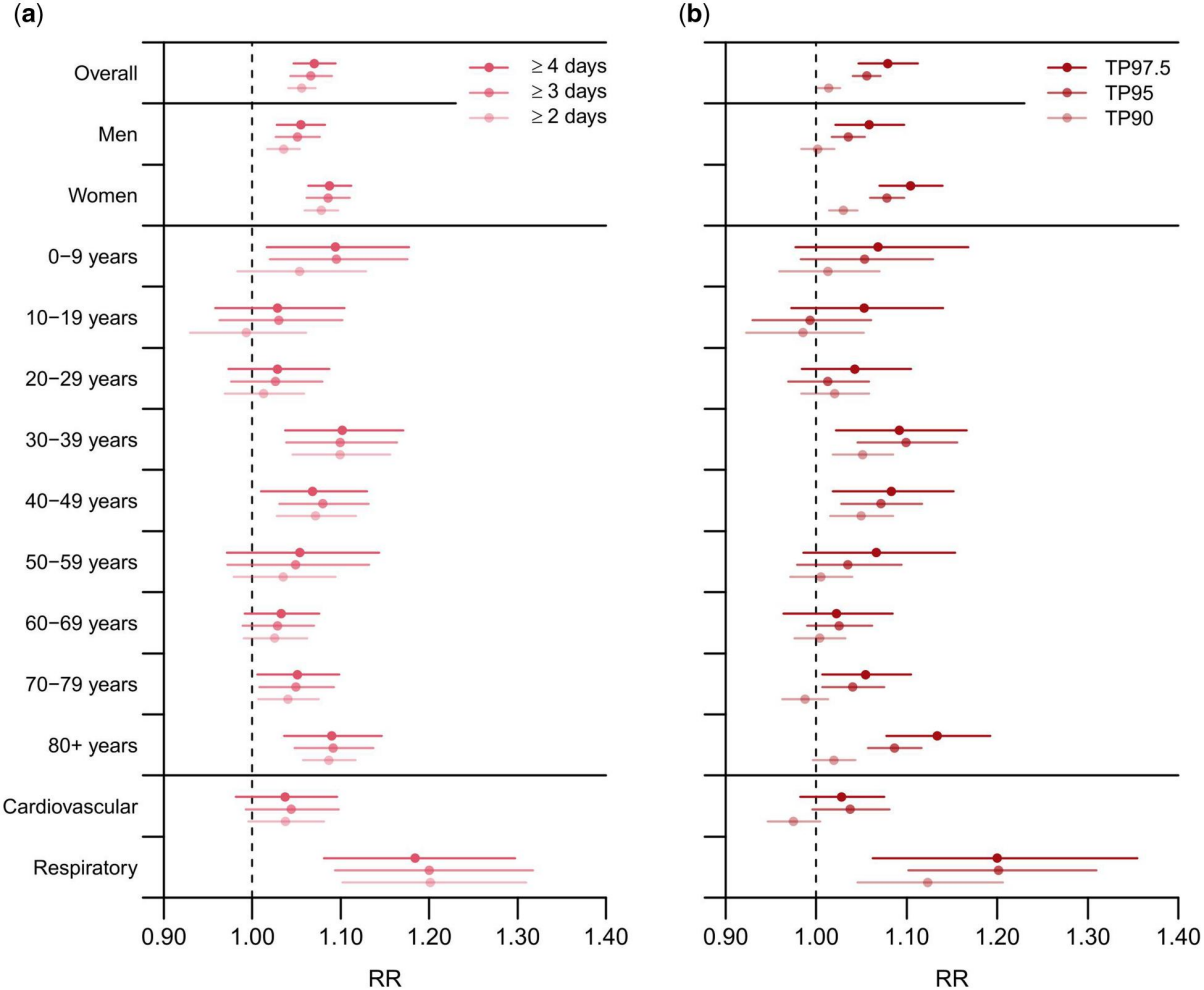
Cumulative risk of ambulance dispatches associated with heatwaves according to different duration (A) and different temperature thresholds (B) in heatwave definitions. Temperature threshold for heat waves definition in panel (A) is 95th temperature percentile. Duration of heatwaves in panel (B) is ≥2 consecutive days. RR, relative risk. TP90, temperature percentile 90. TP95, temperature percentile 95. TP97.5, temperature percentile 97.5. Numerical information is reported in Supplementary Table S5.

Heatwaves had a significant cumulative (lag 0–10 days) added effect on ambulance dispatches in most of the study subgroups (Fig. 4 and Supplementary Table S5). For heatwaves, when the same threshold was used, longer heat waves (≥4 days) had similar heatwave effect estimates as heatwaves with a shorter duration of days (≥2 days) (Fig. 4a). In general, heat waves with higher temperature thresholds had higher effect estimates (Fig. 4b).

**Figure 5.**
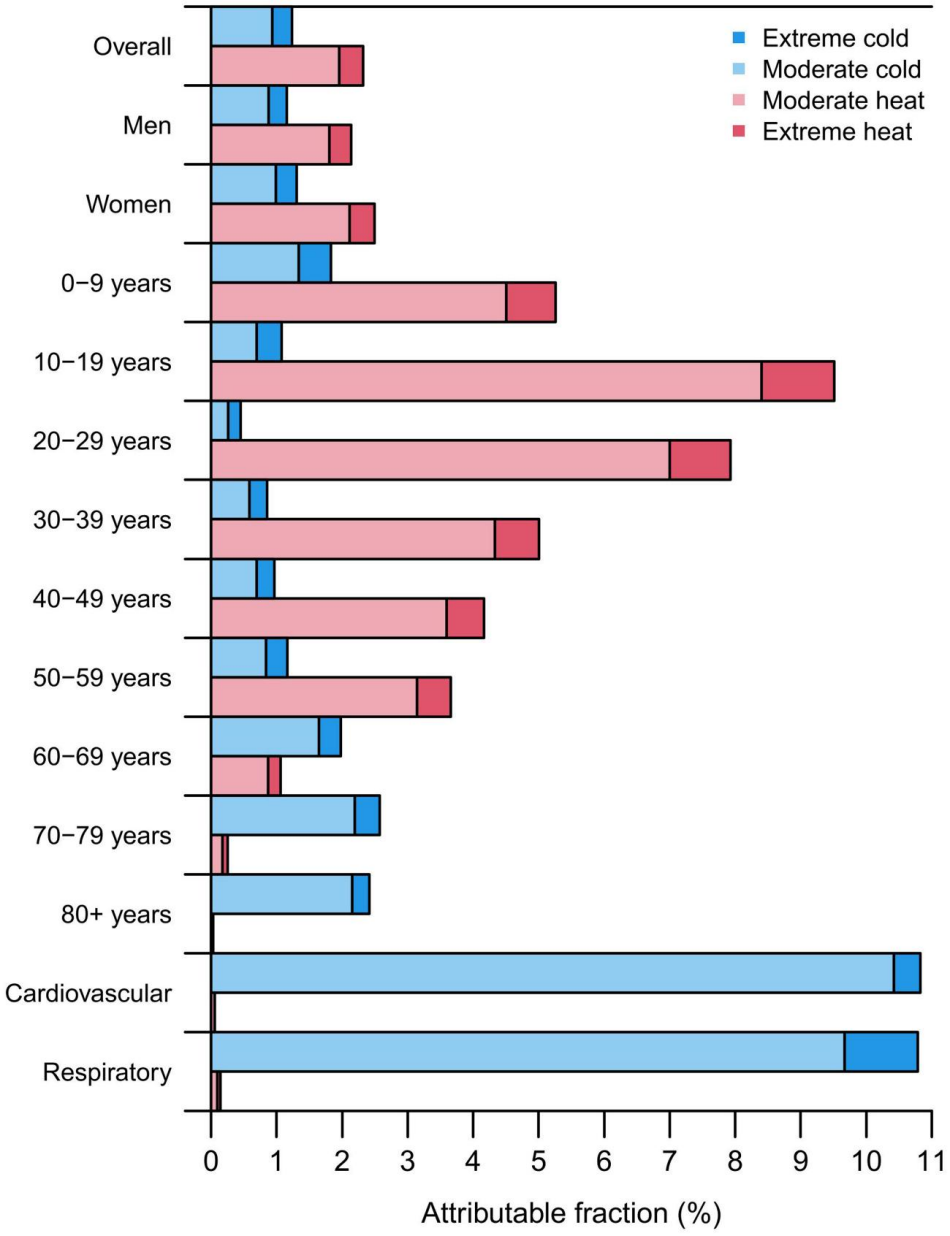
Fraction of ambulance dispatches attributable to moderate and extreme hot and cold temperatures. Extreme and moderate high and low temperatures were defined with the minimum ambulance dispatches temperature and the 2.5th and 97.5th district-specific percentiles of temperature distribution as cut-offs. Numerical information is reported in Supplementary Table S6.

Hot and cold temperatures were responsible for 30 668 (95% eCI: 29 615 to 31 713) annual ambulance dispatches, which was 3.55% (95% eCI: 3.43 to 3.67) of all ambulance dispatches recorded (Fig. 5 and Supplementary Table S6). The contribution of heat [2.32% (2.15 to 2.47)] was predominant and almost double compared with cold [1.23% (1.16 to 1.30)]. This difference was even more pronounced in age groups of <59 years. For instance, the attributable fraction of ambulance dispatches due to heat in young adults (20–29 years) was >17 times greater than the cold-related attributable fraction [7.93% (7.28 to 8.50) vs. 0.45% (0.37 to 0.53)]. The contribution of heat to ambulance dispatches due to cardiovascular and respiratory causes was minor. On the other hand, the cold-attributable fraction exceeded 10% in both cases [10.82% (8.49 to 12.30) for cardiovascular and 10.79% (8.65 to 12.17) for respiratory, respectively].

All sensitivity analyses suggested that the results reported were generally not dependent on modelling assumptions (see Supplementary Table S1 and Supplementary Fig. S2).

## Discussion

This is the first nationwide study of temperature-related ambulance dispatches in Europe, introducing, for the first time, a comprehensive description of age-specific risks for 10-year age groups. Our results showed that there is a substantial risk of increased demand for ambulance services associated with both hot and cold temperatures, with a slightly higher effect observed for heat compared with cold. This risk is particularly pronounced during childhood, adolescence, and early adulthood. The health risks were almost wholly dominated by daily mean temperature whereas the contribution of heat waves was secondary and significant only in certain subpopulations. In addition, our results demonstrate that temperature is responsible for a substantial fraction of ambulance dispatches. Most of this ambulance dispatch burden was caused by heat, accounting for ≤9.51% among the subpopulation aged 10–19 years.

To date, only a few studies have explored the relationship between ambient temperatures and ambulance dispatch data. In contrast to our results, studies from Japan,^20^^,^^25^ Brisbane (Australia),^19^ and 15 cities and counties within Taiwan^15^ have reported a higher risk associated with cold temperatures compared with heat. However, a direct comparison with current literature needs to be interpreted with caution, given that most of the few available studies have focused on urban populations^16^^,^^17^^,^^19^^,^^21^^,^^23^^,^^24^^,^^33^ or were limited to specific regions with distinct climate and socioeconomic conditions.^15^^,^^18^^,^^20^^,^^25^ Additionally, the majority of studies have primarily examined the effects of hot temperatures only.^21^^,^^22^^,^^33^

Although existing evidence suggests a higher vulnerability to heat among women compared with men, primarily mediated by sex-specific physiological differences in thermoregulation and socioeconomic factors,^6^^,^^8^^,^^11^^,^^34–36^ our study found similar risks between both sexes. In principle, the physiological mechanisms underlying heat vulnerability seem to be largely related to cardiovascular and respiratory adverse health outcomes,^11^ which represent only a portion (19.7%) of all ambulance dispatches, and therefore the overall effect may be masked. On the other hand, heatwaves had a greater added effect on ambulance dispatches among women compared with men. This indicates that sex-related differences are even amplified after prolonged exposure to extreme heat, which is in alignment with the mortality studies after heatwave episodes.^36^

Age has also been linked to physiological differences in thermoregulation, with extensive evidence of the greatest health effects in older individuals.^6^^,^^8^^,^^11^ However, an accurate comparison with previous studies is limited, as, to the best of our knowledge, this is the first analysis to quantify detailed age-specific risks by 10-year age groups. Conversely, in this study, we observed the highest risk of ambulance dispatches for both hot and cold temperatures among the youngest age categories. This finding supports the conclusion that climate change and associated temperature increase constitute detrimental challenge for the health and wellbeing of young populations.^37^ Limited ability to thermoregulate and a higher risk of dehydration or fever are presumable explanations that might play a role in babies and during childhood.^37–40^ With increasing age towards adolescence and young adulthood, the possible range of explanations seems to be shifted more to behavioural factors. Children and adolescents tend to spend more time outdoors, leading to increased exposure to ambient temperatures.^40^ Additionally, warmer weather has been linked to higher trauma rates independently of the effects of seasonal variation and this relationship is stronger in younger individuals than in adults.^41^ Furthermore, both cold and hot ambient temperatures increase the risk of car accidents,^42^ with young adults being particularly susceptible.^43^ Moreover, young workers have been shown to be more vulnerable to heat-related^44–46^ and cold-related^45^ occupational injuries, possibly due to an inclination towards more physically demanding work in outdoor extreme weather conditions and lack of experience.^46^ Additionally, there is evidence that elevated temperatures are associated with increased alcohol consumption and drug use, leading to subsequent health disorders,^47–49^ which, in retrospect, can also lead to impaired thermoregulatory response.^50^

The cause-specific analysis revealed that heat does not result in a significant increase in ambulance dispatches for cardiovascular or respiratory causes. This finding aligns with previous literature on cardiovascular diseases,^16^^,^^19^^,^^22^^,^^25^^,^^51^ although evidence regarding respiratory diseases remains inconclusive.^19^^,^^20^^,^^22^^,^^25^ Concurrently, given the limited effects of heat on ambulance dispatches among the oldest age categories, we assume that many heat-related cardiorespiratory health impacts may occur suddenly, leading to death before any medical attention or even before ambulance callout.^11^ In addition, it is worth noting that elderly people often live alone^52^ and may be less familiar with using a cell phone, which could complicate their ability to seek medical attention.^53^ Simultaneously, a substantial number of elderly individuals reside in retirement homes with medical care (>15% people in the age category 86+ years),^54^ thereby reducing the need for ambulances. Conversely, heatwaves had an additional effect on ambulance dispatches for respiratory causes and among the oldest age categories, which may indicate that prolonged periods of extreme temperatures may affect wider populations and thus increase the risk of ambulance dispatches.

In contrast to mortality studies,^8^^,^^55^^,^^56^ we saw that most of the ambulance dispatches burden was attributed to days that were warmer than the optimum temperature [2.32% (95% eCI: 2.15 to 2.47)] compared with days that were colder than the optimum temperature [1.23% (1.16 to 1.30)]. We identified younger individuals as the most vulnerable subgroup, with a substantial effect of heat on ambulance dispatches. Nearly every tenth ambulance dispatch among individuals aged 10–19 years was attributed to heat. This might be a reason for concern given that a progressive increase in temperatures of between 1.7°C and 4.5°C (based on the Representative Concentration Pathway scenario) is projected to occur during the twenty-first century.^56^ These findings emphasize the importance of implementing prevention plans that should also target younger populations and the need for a long-term strategy to prepare emergency medical services for the anticipated challenges posed by rising temperatures.

There are some study limitations worth acknowledging. First, in our models, we were not able to adjust for air pollution or influenza epidemics due to data unavailability. Nevertheless, air pollution is more likely to be a mediator rather than a confounder in the relationship between temperature and health outcomes.^11^ Moreover, current literature shows that accounting for air pollutants had either no or only a minimal impact on the relationship between temperature and health outcomes.^55^^,^^57–59^ Second, although our results suggest low spatial heterogeneity in attributable risk for both heat and cold, the findings cannot be interpreted as pan-European representative due to differences in population characteristics and climates. This limitation can be addressed in future research by extending the dataset to other countries.

The findings of this study have important implications for healthcare system preparedness and provide important evidence for better management of existing medical and emergency resources (e.g. short-term shift plans during extreme temperature events, long-term capacity expansion due to rising temperatures, adequate medical material distribution targeting the most vulnerable populations). The study further provides key information for climate change health adaptation policies and the projections of climate change health impacts expand the scope beyond commonly studied health outcomes such as mortality and hospital admissions. While the majority of epidemiological studies have explored temperature-related mortality and hospital admissions, there is still limited evidence about the impacts of rising temperatures on other health outcomes and associated societal and economic consequences. Compared with mortality or hospital admissions, ambulance dispatches offer valuable and timely health information on early signs of the impacts of extreme and moderate temperatures.^21^^,^^60^ While ambulance dispatches primarily serve as indicators of non-fatal and acute health events, they play a crucial role in identifying vulnerable population subgroups, such as children, adolescents, and young adults. The results suggest that public health policies and adaptation strategies need to be extended and refocused to consider a wider range of vulnerable subgroups, which are often overlooked. Furthermore, our study also provides an important platform for emergency medical services and underscores the need to develop and/or strengthen heat surveillance systems and implement long-term adaptation strategies.

## Ethics approval

The data used are anonymized and aggregated, and thus do not include any personal data. Therefore, ethics approval is not applicable.

## Supplementary Material

dyaf051_Supplementary_Data

## Data Availability

All the data used in this study are routinely collected and contain no information about specific people. Health data can be requested through the Institute of Health Information and Statistics of the Czech Republic (https://www.uzis.cz/index-en.php). Meteorological data can be freely obtained from the E-OBS gridded dataset (https://doi.org/10.24381/cds.151d3ec6).
