# Supplementary material for: Short-term effect of temperature on cause-specific, sex-specific, and age-specific ambulance dispatches in Czechia: a nationwide time-series analysis
Source: Int J Epidemiol. 2025 May 28;54(3):dyaf051. doi: 10.1093/ije/dyaf051 (PMC12117535; doi:10.1093/ije/dyaf051)
Supplement: dyaf051_Supplementary_Data [file dyaf051_supplementary_data.docx]

***Supplementary Material***

**Short-term effect of temperature on cause-specific, sex-specific and age-specific ambulance dispatches in Czechia: a nationwide time-series analysis**

*Tomáš Janoš^1,2^, Joan Ballester^2^, Raúl F. Méndez-Turrubiates^2^, Pavel Čupr^1^*, Hicham Achebak^2,3^*

^1^RECETOX, Faculty of Science, Masaryk University, Brno, Czech Republic.

^2^ISGlobal, Barcelona, Spain.

^3^Inserm, France Cohortes, Paris, France.

*Corresponding author:

Assoc. Prof. Pavel Čupr,

RECETOX Centre, Faculty of Science, Masaryk University,

Kamenice 753/5, Pavilon A29, 62500 Brno, Czech Republic,

[*pavel.cupr@recetox.muni.cz*](mailto:pavel.cupr@recetox.muni.cz)*, +420 549 493 511*

**Table of contents**

[Table S1. Different choices of model configuration 3](#_Toc164698060)

[Table S2. Number of ambulance dispatches by sex, age, specific diseases and year of the study. 4](#_Toc164698061)

[Table S3. Descriptive statistics of the daily mean population-weighted temperature (°C) during the study period. 5](#_Toc164698062)

[Figure S1. Spatial distribution of the daily mean population-weighted temperature during the study period between October and April (A) and between May and September (B). 6](#_Toc164698063)

[Table S4. Relative risk (RR) of ambulance dispatches associated with cold (1^st^ temperature percentile) and heat (99^th^ temperature percentile) vs. the minimum ambulance dispatches temperature (MADT). 7](#_Toc164698064)

[Table S5. Cumulative effects of heat waves on ambulance dispatches over lag 0-10 days expressed as relative risk (RR) according to different heat wave definitions. 8](#_Toc164698065)

[Table S6. Cold- and heat-attributable fraction (AF%) of ambulance dispatches with 95% empirical confidence intervals. 9](#_Toc164698066)

[Figure S2. Sensitivity analysis on ambulance dispatches fraction attributable to non-optimum, cold and hot temperatures by varying modelling choices. 10](#_Toc164698067)

# Table S1. Different choices of model configuration

|  | Model configuration | Akaike Information Criterion | RR at TP01 (95% eCI) | RR at TP99 (95% eCI) |
| --- | --- | --- | --- | --- |
| **Main Model** | Exposure-Response: Knot at Percentile P50  Lag-Response: 3 internal knots placed at equally spaced values in the log scale | 1,770,813 | 1.138 (1.122, 1.153) | 1.155 (1.128, 1.182) |
| **Alternative Models** |  |  |  |  |
| Model 1 | Exposure-Response: Knot at Percentile P75  Lag-Response: 3 internal knots placed at equally spaced values in the log scale | 1,770,865 | 1.134 (1.118, 1.150) | 1.159 (1.133, 1.186) |
| Model 2 | Exposure-Response: Knots at Percentiles P25, P75  Lag-Response: 3 internal knots placed at equally spaced values in the log scale | 1,771,062 | 1.131 (1.113, 1.150) | 1.167 (1.143, 1.192) |
| Model 3 | Exposure-Response: Knots at Percentiles P25, P50  Lag-Response: 3 internal knots placed at equally spaced values in the log scale | 1,771,077 | 1.133 (1.115, 1.151) | 1.173 (1.149, 1.197) |
| Model 4 | Exposure-Response: Knots at Percentiles P10, P50, P90  Lag-Response: 3 internal knots placed at equally spaced values in the log scale | 1,771,179 | 1.131 (1.113, 1.150) | 1.170 (1.144, 1.196) |
| Model 5 | Exposure-Response: Knots at Percentiles P10, P75, P90  Lag-Response: 3 internal knots placed at equally spaced values in the log scale | 1,771,313 | 1.131 (1.112, 1.150) | 1.160 (1.134, 1.188) |
| Model 6 | Exposure-Response: Knot at Percentile P50  Lag-Response: 2 internal knots placed at equally spaced values in the log scale | 1,771,128 | 1.146 (1.130, 1.163) | 1.133 (1.107, 1.160) |

RR, relative risk

TP01, temperature percentile 1

TP99, temperature percentile 99

95% eCI, 95% empirical Confidence Interval

# Table S2. Number of ambulance dispatches by sex, age, specific diseases and year of the study.

|  | 2010-2019 |  | 2010 |  | 2011 |  | 2012 |  | 2013 |  | 2014 |  | 2015 |  | 2016 |  | 2017 |  | 2018 |  | 2019 |
| --- | --- | --- | --- | --- | --- | --- | --- | --- | --- | --- | --- | --- | --- | --- | --- | --- | --- | --- | --- | --- | --- |
| **Overall** | 8634643 (100%) |  | 722621 |  | 745192 |  | 793071 |  | 828343 |  | 858468 |  | 914513 |  | 906509 |  | 920629 |  | 976067 |  | 969230 |
| **Men** | 4337784 (50.2%) |  | 366038 |  | 378227 |  | 401118 |  | 416772 |  | 429304 |  | 458410 |  | 451653 |  | 459775 |  | 489335 |  | 487155 |
| **Women** | 4296859 (49.8%) |  | 356583 |  | 366965 |  | 391956 |  | 411571 |  | 429164 |  | 456103 |  | 454856 |  | 460854 |  | 486732 |  | 482075 |
| **0-9 years** | 363758 (4.2%) |  | 29810 |  | 32608 |  | 34284 |  | 35996 |  | 36524 |  | 39086 |  | 38255 |  | 38394 |  | 39555 |  | 39242 |
| **10-19 years** | 377849 (4.4%) |  | 33767 |  | 34974 |  | 35601 |  | 34913 |  | 36437 |  | 37641 |  | 38897 |  | 39848 |  | 42561 |  | 43210 |
| **20-29 years** | 699318 (8.1%) |  | 63041 |  | 66419 |  | 69594 |  | 72143 |  | 73812 |  | 74450 |  | 71988 |  | 69592 |  | 70619 |  | 67660 |
| **30-39 years** | 752835 (8.7%) |  | 68207 |  | 70991 |  | 74356 |  | 76416 |  | 77912 |  | 78475 |  | 76954 |  | 75969 |  | 78099 |  | 75456 |
| **40-49 years** | 778188 (9%) |  | 63321 |  | 65708 |  | 70355 |  | 73662 |  | 76972 |  | 80919 |  | 82039 |  | 83341 |  | 90217 |  | 91654 |
| **50-59 years** | 901419 (10.4%) |  | 86780 |  | 86161 |  | 87449 |  | 89086 |  | 90029 |  | 93672 |  | 91352 |  | 90291 |  | 93931 |  | 92668 |
| **60-69 years** | 1385636 (16%) |  | 116181 |  | 120553 |  | 131239 |  | 137270 |  | 140182 |  | 151464 |  | 147048 |  | 145817 |  | 150725 |  | 145157 |
| **70-79 years** | 1572001 (18.2%) |  | 120363 |  | 120646 |  | 129581 |  | 138138 |  | 146889 |  | 164021 |  | 168589 |  | 180680 |  | 199438 |  | 203656 |
| **80+ years** | 1803639 (20.9%) |  | 141151 |  | 147132 |  | 160608 |  | 170719 |  | 179711 |  | 194785 |  | 191387 |  | 196697 |  | 210922 |  | 210527 |
| **Cardiovascular** | 1383740 (16%) |  | 137235 |  | 137112 |  | 143563 |  | 139589 |  | 140393 |  | 144883 |  | 138659 |  | 135151 |  | 135137 |  | 132018 |
| **Respiratory** | 315690 (3.7%) |  | 29252 |  | 30436 |  | 31044 |  | 34207 |  | 30802 |  | 33808 |  | 31201 |  | 31547 |  | 33086 |  | 30307 |

# Table S3. Descriptive statistics of the daily mean population-weighted temperature (°C) during the study period.

|  | 2010 |  | 2011 |  | 2012 |  | 2013 |  | 2014 |  | 2015 |  | 2016 |  | 2017 |  | 2018 |  | 2019 |
| --- | --- | --- | --- | --- | --- | --- | --- | --- | --- | --- | --- | --- | --- | --- | --- | --- | --- | --- | --- |
| **Annual** |  |  |  |  |  |  |  |  |  |  |  |  |  |  |  |  |  |  |  |
| mean | 7.55 |  | 8.93 |  | 8.75 |  | 8.37 |  | 9.88 |  | 9.90 |  | 9.11 |  | 9.07 |  | 10.16 |  | 10.07 |
| median | 8.29 |  | 9.27 |  | 8.58 |  | 8.66 |  | 10.44 |  | 9.29 |  | 8.39 |  | 9.28 |  | 11.24 |  | 9.65 |
| minimum | -19.48 |  | -13.85 |  | -19.03 |  | -12.68 |  | -13.74 |  | -7.44 |  | -15.17 |  | -17.68 |  | -14.86 |  | -10.05 |
| maximum | 29.25 |  | 28.49 |  | 29.10 |  | 30.31 |  | 27.95 |  | 30.62 |  | 27.67 |  | 30.01 |  | 29.61 |  | 29.98 |
| IQR | 13.99 |  | 13.38 |  | 13.22 |  | 13.60 |  | 10.65 |  | 11.82 |  | 13.42 |  | 12.69 |  | 15.32 |  | 12.03 |
| **Hot months** |  |  |  |  |  |  |  |  |  |  |  |  |  |  |  |  |  |  |  |
| mean | 15.60 |  | 16.20 |  | 16.64 |  | 15.83 |  | 15.91 |  | 17.13 |  | 16.88 |  | 16.70 |  | 18.22 |  | 16.98 |
| median | 15.15 |  | 16.22 |  | 16.66 |  | 15.61 |  | 15.78 |  | 16.29 |  | 17.06 |  | 16.79 |  | 18.53 |  | 17.20 |
| minimum | 3.66 |  | -0.99 |  | 3.58 |  | 4.03 |  | 1.92 |  | 5.06 |  | 3.86 |  | 1.11 |  | 3.75 |  | 1.53 |
| maximum | 29.25 |  | 28.49 |  | 29.10 |  | 30.31 |  | 27.95 |  | 30.62 |  | 27.67 |  | 30.01 |  | 29.61 |  | 29.98 |
| IQR | 6.82 |  | 5.12 |  | 5.98 |  | 6.80 |  | 5.54 |  | 8.00 |  | 4.88 |  | 6.60 |  | 5.49 |  | 7.02 |
| **Cold months** |  |  |  |  |  |  |  |  |  |  |  |  |  |  |  |  |  |  |  |
| mean | 1.74 |  | 3.68 |  | 3.09 |  | 2.99 |  | 5.53 |  | 4.69 |  | 3.54 |  | 3.57 |  | 4.35 |  | 5.08 |
| median | 2.10 |  | 3.13 |  | 3.86 |  | 2.26 |  | 5.06 |  | 4.13 |  | 3.57 |  | 3.88 |  | 3.53 |  | 4.88 |
| minimum | -19.48 |  | -13.85 |  | -19.03 |  | -12.68 |  | -13.74 |  | -7.44 |  | -15.17 |  | -17.68 |  | -14.86 |  | -10.05 |
| maximum | 20.29 |  | 19.46 |  | 24.05 |  | 20.12 |  | 17.33 |  | 18.37 |  | 19.45 |  | 16.97 |  | 22.25 |  | 19.83 |
| IQR | 10.53 |  | 7.72 |  | 7.31 |  | 7.98 |  | 7.03 |  | 6.30 |  | 6.49 |  | 7.40 |  | 9.48 |  | 7.10 |

Cold months of the year defined as October-April. Hot months of the year defined as May-September.

# Figure S1. Spatial distribution of the daily mean population-weighted temperature during the study period between October and April (A) and between May and September (B).

**
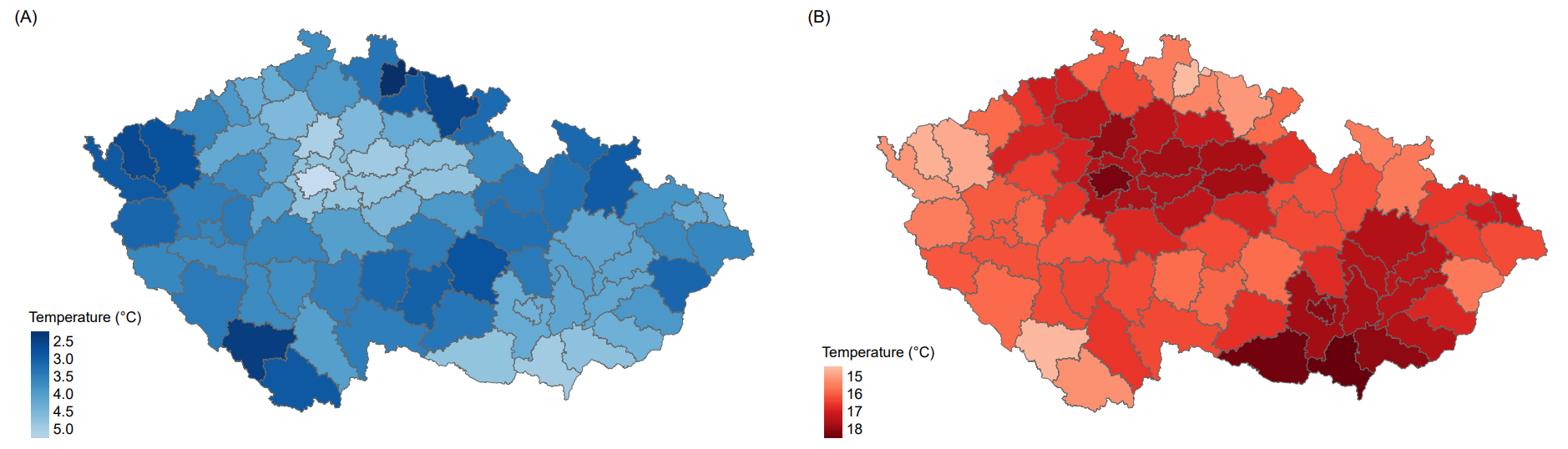
**

# Table S4. Relative risk (RR) of ambulance dispatches associated with cold (1^st^ temperature percentile) and heat (99^th^ temperature percentile) vs. the minimum ambulance dispatches temperature (MADT).

|  | Cold | | |  | Heat | | |
| --- | --- | --- | --- | --- | --- | --- | --- |
|  | RR |  | 95% eCI |  | RR |  | 95% eCI |
| **Overall** | 1.138 |  | 1.122-1.153 |  | 1.155 |  | 1.128-1.182 |
| **Men** | 1.129 |  | 1.107-1.151 |  | 1.147 |  | 1.121-1.173 |
| **Women** | 1.149 |  | 1.124-1.174 |  | 1.167 |  | 1.136-1.199 |
| **0-9 years** | 1.235 |  | 1.164-1.31 |  | 1.395 |  | 1.288-1.51 |
| **10-19 years** | 1.195 |  | 1.132-1.261 |  | 1.692 |  | 1.563-1.831 |
| **20-29 years** | 1.088 |  | 1.046-1.132 |  | 1.457 |  | 1.357-1.564 |
| **30-39 years** | 1.124 |  | 1.077-1.174 |  | 1.312 |  | 1.241-1.388 |
| **40-49 years** | 1.126 |  | 1.076-1.18 |  | 1.258 |  | 1.191-1.328 |
| **50-59 years** | 1.145 |  | 1.095-1.197 |  | 1.239 |  | 1.169-1.312 |
| **60-69 years** | 1.144 |  | 1.104-1.186 |  | 1.078 |  | 1.032-1.127 |
| **70-79 years** | 1.179 |  | 1.141-1.219 |  | 1.03 |  | 1.001-1.059 |
| **80+ years** | 1.119 |  | 1.073-1.167 |  | 1.003 |  | 0.986-1.02 |
| **Cardiovascular** | 1.181 |  | 1.097-1.272 |  | 1.025 |  | 1.013-1.038 |
| **Respiratory** | 1.466 |  | 1.336-1.608 |  | 1.006 |  | 0.962-1.052 |

RR, relative risk

95% eCI, 95% empirical Confidence Interval

# Table S5. Cumulative effects of heat waves on ambulance dispatches over lag 0-10 days expressed as relative risk (RR) according to different heat wave definitions.

|  | **Temperature threshold = 95^th^ temperature percentile** | | | | |  | **Heat wave duration ≥2 days** | | | | |  |
| --- | --- | --- | --- | --- | --- | --- | --- | --- | --- | --- | --- | --- |
|  | ≥2 days |  | ≥3 days |  | ≥4 days |  | TP90 |  | TP95 |  | TP97.5 |  |
| **Overall** | 1.056 (1.041-1.071) |  | 1.066 (1.043-1.09) |  | 1.07 (1.046-1.094) |  | 1.014 (1.001-1.026) |  | 1.056 (1.041-1.071) |  | 1.079 (1.047-1.112) |  |
| **Men** | 1.035 (1.017-1.054) |  | 1.051 (1.026-1.076) |  | 1.055 (1.028-1.083) |  | 1.002 (0.983-1.02) |  | 1.035 (1.017-1.054) |  | 1.058 (1.021-1.097) |  |
| **Women** | 1.078 (1.059-1.097) |  | 1.086 (1.062-1.11) |  | 1.087 (1.063-1.112) |  | 1.03 (1.014-1.046) |  | 1.078 (1.059-1.097) |  | 1.104 (1.07-1.139) |  |
| **0-9 years** | 1.053 (0.983-1.129) |  | 1.095 (1.02-1.175) |  | 1.094 (1.017-1.177) |  | 1.013 (0.959-1.07) |  | 1.053 (0.983-1.129) |  | 1.068 (0.977-1.168) |  |
| **10-19 years** | 0.993 (0.93-1.061) |  | 1.03 (0.963-1.102) |  | 1.028 (0.958-1.104) |  | 0.985 (0.922-1.053) |  | 0.993 (0.93-1.061) |  | 1.053 (0.972-1.14) |  |
| **20-29 years** | 1.013 (0.969-1.058) |  | 1.026 (0.976-1.079) |  | 1.028 (0.973-1.087) |  | 1.02 (0.984-1.058) |  | 1.013 (0.969-1.058) |  | 1.043 (0.984-1.105) |  |
| **30-39 years** | 1.099 (1.046-1.156) |  | 1.099 (1.039-1.164) |  | 1.102 (1.037-1.171) |  | 1.051 (1.018-1.085) |  | 1.099 (1.046-1.156) |  | 1.092 (1.022-1.166) |  |
| **40-49 years** | 1.071 (1.028-1.117) |  | 1.08 (1.031-1.131) |  | 1.068 (1.01-1.13) |  | 1.05 (1.015-1.085) |  | 1.071 (1.028-1.117) |  | 1.083 (1.019-1.151) |  |
| **50-59 years** | 1.035 (0.979-1.094) |  | 1.049 (0.972-1.132) |  | 1.054 (0.971-1.144) |  | 1.005 (0.971-1.04) |  | 1.035 (0.979-1.094) |  | 1.066 (0.986-1.154) |  |
| **60-69 years** | 1.025 (0.99-1.062) |  | 1.028 (0.989-1.07) |  | 1.033 (0.991-1.076) |  | 1.004 (0.976-1.032) |  | 1.025 (0.99-1.062) |  | 1.022 (0.964-1.084) |  |
| **70-79 years** | 1.04 (1.007-1.075) |  | 1.049 (1.008-1.092) |  | 1.051 (1.006-1.098) |  | 0.988 (0.963-1.013) |  | 1.04 (1.007-1.075) |  | 1.055 (1.006-1.105) |  |
| **80+ years** | 1.086 (1.057-1.116) |  | 1.091 (1.048-1.137) |  | 1.09 (1.036-1.147) |  | 1.019 (0.996-1.043) |  | 1.086 (1.057-1.116) |  | 1.134 (1.078-1.192) |  |
| **Cardiovascular** | 1.037 (0.996-1.081) |  | 1.044 (0.993-1.098) |  | 1.037 (0.982-1.096) |  | 0.975 (0.946-1.004) |  | 1.037 (0.996-1.081) |  | 1.028 (0.983-1.075) |  |
| **Respiratory** | 1.201 (1.102-1.31) |  | 1.2 (1.093-1.317) |  | 1.184 (1.081-1.297) |  | 1.123 (1.046-1.206) |  | 1.201 (1.102-1.31) |  | 1.2 (1.063-1.355) |  |

TP90, temperature percentile 90. TP95, temperature percentile 95. TP97.5, temperature percentile 97.5.

# Table S6. Cold- and heat-attributable fraction (AF%) of ambulance dispatches with 95% empirical confidence intervals.

|  |  |  | Cold days | | |  | Heat days | | |
| --- | --- | --- | --- | --- | --- | --- | --- | --- | --- |
|  | Total |  | Extreme cold |  | Moderate cold |  | Moderate heat |  | Extreme heat |
| **Overall** | 3.55% (3.43-3.67) |  | 0.3% (0.29-0.31) |  | 0.94% (0.87-0.99) |  | 1.96% (1.81-2.09) |  | 0.36% (0.34-0.38) |
| **Men** | 3.3% (3.14-3.44) |  | 0.28% (0.26-0.3) |  | 0.88% (0.81-0.95) |  | 1.81% (1.73-1.88) |  | 0.34% (0.32-0.35) |
| **Women** | 3.8% (3.66-3.91) |  | 0.32% (0.3-0.34) |  | 0.99% (0.88-1.1) |  | 2.11% (1.92-2.28) |  | 0.39% (0.36-0.4) |
| **0-9 years** | 7.09% (6.81-7.33) |  | 0.5% (0.47-0.52) |  | 1.33% (1.22-1.44) |  | 4.51% (4.27-4.74) |  | 0.75% (0.72-0.78) |
| **10-19 years** | 10.58% (10.35-10.8) |  | 0.38% (0.36-0.39) |  | 0.69% (0.64-0.75) |  | 8.41% (8.2-8.63) |  | 1.11% (1.08-1.12) |
| **20-29 years** | 8.38% (7.76-8.88) |  | 0.2% (0.17-0.22) |  | 0.26% (0.2-0.31) |  | 7% (6.41-7.53) |  | 0.93% (0.86-0.97) |
| **30-39 years** | 5.86% (5.56-6.15) |  | 0.27% (0.23-0.3) |  | 0.58% (0.44-0.7) |  | 4.34% (4.14-4.52) |  | 0.67% (0.64-0.7) |
| **40-49 years** | 5.13% (4.83-5.33) |  | 0.27% (0.22-0.31) |  | 0.7% (0.49-0.89) |  | 3.59% (3.23-3.95) |  | 0.57% (0.54-0.6) |
| **50-59 years** | 4.82% (4.39-5.21) |  | 0.33% (0.27-0.36) |  | 0.84% (0.64-1) |  | 3.14% (2.77-3.48) |  | 0.52% (0.46-0.56) |
| **60-69 years** | 3.04% (2.46-3.45) |  | 0.33% (0.3-0.36) |  | 1.65% (1.12-2.1) |  | 0.87% (0.55-1.14) |  | 0.19% (0.13-0.22) |
| **70-79 years** | 2.82% (2.71-2.92) |  | 0.38% (0.37-0.39) |  | 2.2% (2.08-2.31) |  | 0.18% (0.13-0.22) |  | 0.07% (0.06-0.08) |
| **80+ years** | 2.44% (1.28-3.43) |  | 0.26% (0.22-0.3) |  | 2.15% (0.91-3.2) |  | 0.02% (-0.01-0.04) |  | 0.01% (0-0.02) |
| **Cardiovascular** | 10.88% (8.64-12.44) |  | 0.4% (0.3-0.45) |  | 10.43% (8.19-11.85) |  | 0.05% (-0.12-0.21) |  | 0% (-0.01-0.01) |
| **Respiratory** | 10.93% (8.95-12.33) |  | 1.12% (1.04-1.17) |  | 9.67% (7.61-11) |  | 0.1% (0.01-0.17) |  | 0.04% (0.02-0.07) |

Heat and cold days were defined as days with temperatures higher and lower than the minimum ambulance dispatches temperature. These days were further separated into moderate and extreme parts by defining extreme heat (cold) as temperatures higher (lower) than the 97.5^th^ (2.5^th^) district-specific percentile.


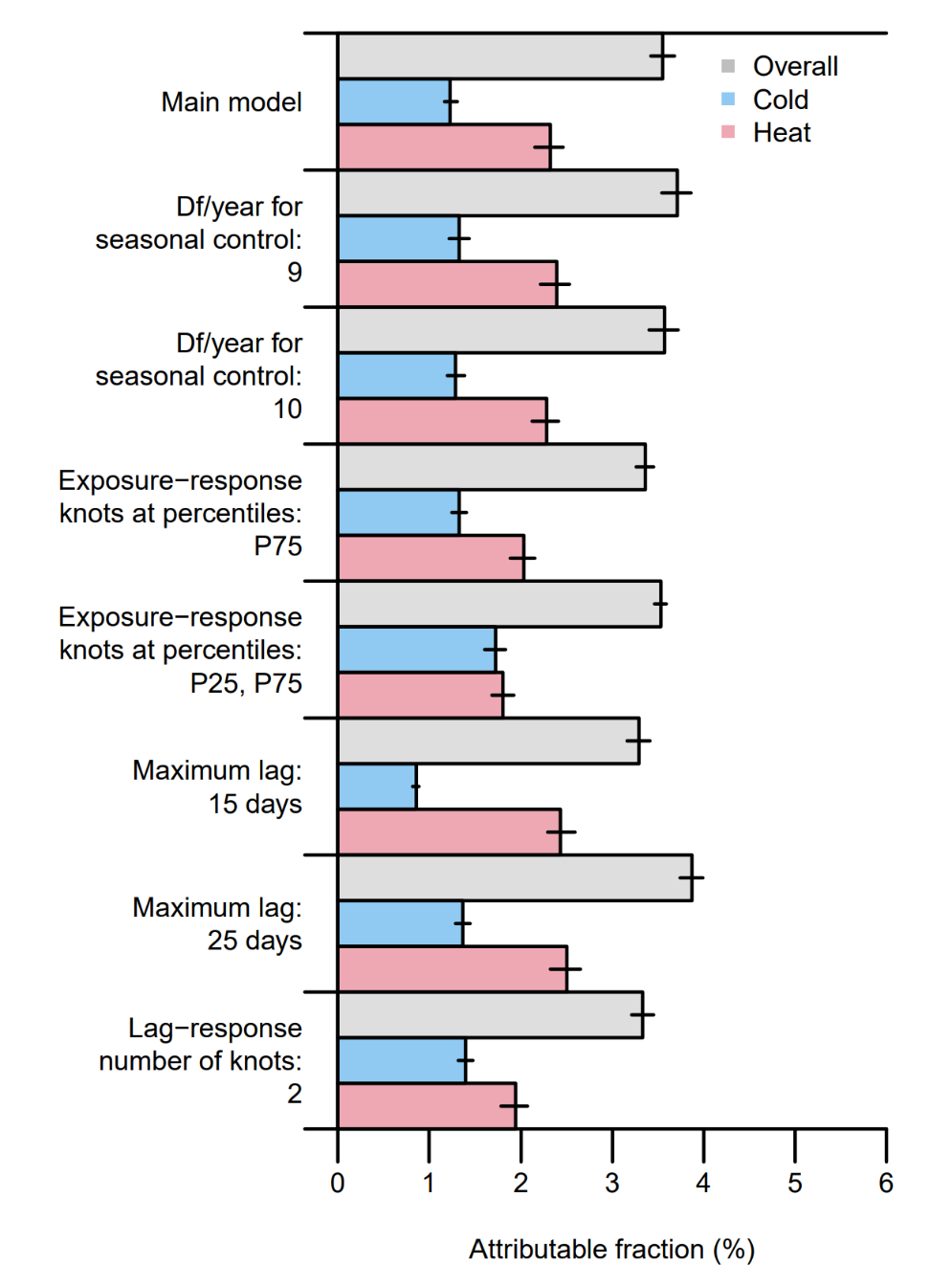
Figure S2. Sensitivity analysis on ambulance dispatches fraction attributable to non-optimum, cold and hot temperatures by varying modelling choices. Degrees of freedom for seasonal trend, the number and placement of knots for exposure-response function, the number of knots in the lag-response function, lag period.

Error bars represent the 95% empirical CI.
